# How Wind Shear Affects Trade‐wind Cumulus Convection

**DOI:** 10.1029/2020MS002183

**Published:** 2020-11-28

**Authors:** K. C. Helfer, L. Nuijens, S. R. de Roode, A. P. Siebesma

**Affiliations:** ^1^ Department of Geoscience and Remote Sensing Delft University of Technology Delft Netherlands; ^2^ Royal Netherlands Meteorological Institute (KNMI) De Bilt Netherlands

**Keywords:** shallow convection, wind shear, cumulus, trade wind, large‐eddy simulation

## Abstract

Motivated by an observed relationship between marine low cloud cover and surface wind speed, this study investigates how vertical wind shear affects trade‐wind cumulus convection, including shallow cumulus and congestus with tops below the freezing level. We ran large‐eddy simulations for an idealized case of trade‐wind convection using different vertical shears in the zonal wind. Backward shear, whereby surface easterlies become upper westerlies, is effective at limiting vertical cloud development, which leads to a moister, shallower, and cloudier trade‐wind layer. Without shear or with forward shear, shallow convection tends to deepen more, but clouds tops are still limited under forward shear. A number of mechanisms explain the observed behavior: First, shear leads to different surface wind speeds and, in turn, surface heat and moisture fluxes due to momentum transport, whereby the weakest surface wind speeds develop under backward shear. Second, a forward shear profile in the subcloud layer enhances moisture aggregation and leads to larger cloud clusters, but only on large domains that generally support cloud organization. Third, any absolute amount of shear across the cloud layer limits updraft speeds by enhancing the downward oriented pressure perturbation force. Backward shear—the most typical shear found in the winter trades—can thus be argued a key ingredient at setting the typical structure of the trade‐wind layer.

## Key Points

- Shear in the zonal wind influences cloud‐top heights via the effect of momentum transport on the surface wind and surface fluxes
- Backward shear (surface easterlies turn westerlies) lowers cloud tops and shallows and moistens the trade‐wind layer
- Any absolute amount of wind shear limits in‐cloud updraft speeds and enhances low‐level cloud fraction

## 1 Introduction

In light of the uncertain role of trade‐wind cumulus clouds in setting the cloud feedback in climate change, there is widespread interest in understanding the behavior of these clouds, the different ways they interact with their environment, and how this changes in response to global warming (e.g., Bony & Dufresne, 2005; Bony et al., 2013; Vial et al., 2017). Trade‐wind cumuli are found in regions characterized by the trade winds, yet we understand relatively little about how they depend on the structure of the trade wind, compared to how they depend on temperature and moisture. Some studies have investigated the influence of the wind speed on low clouds in the trades and revealed that surface wind speed is one of the better predictors of low cloud amount (e.g., Brueck et al., 2015; Klein et al., 2017; Nuijens & Stevens, 2012). But it is unclear how much the wind shear plays a role in observed cloud amount‐wind speed relationships, as one might expect both wind speed and wind shear to increase with larger meridional temperature gradients throughout the lower troposphere when assuming geostrophic and thermal wind balance. Furthermore, little work has concentrated on the influence of wind shear on convection, other than its role in increasing the amount of projected cloud cover.

From studies of deep convection we know that wind shear can have a number of effects. Shear is effective at organizing deep convective systems into rain bands and squall lines (e.g., Hildebrand, 1998; Parker, 1996; Robe & Emanuel, 2001; Rotunno et al., 1988; Thorpe et al., 1982; Weisman & Rotunno, 2004). At the same time, shear can limit convection during its developing stages (Pastushkov, 1975). A recent paper by Peters et al. (2019) clearly shows how shear reduces updraft speeds in slanted thermals by enhancing the (downward oriented) pressure perturbations. Shear is also argued to inhibit deep convection by “blowing off” cloud tops (e.g., Koren et al., 2010; Sathiyamoorthy et al., 2004), which we interpret as an increase in the cloud surface area that experiences entrainment, which also plays a role in setting updraft buoyancy and updraft speeds.

Malkus (1949) might have been one of the first to mention the effect of shear on shallow convection, noting that the tilting of clouds through shear causes an asymmetry in its turbulence structure with more turbulence on the windward than the leeward side. Through numerous studies we now know that shear helps organize shallow convective clouds in rolls or streets along with the development of coherent moisture and temperature structures in the subcloud layer (e.g., Asai, 1964; Hill, 1968; LeMone & Pennell, 1976; Malkus, 1963; Park et al., 2018). Li et al. (2014) explain how shear over the subcloud layer interacts with the low‐level circulation induced by cold pools to enhance or limit the regeneration of convective cells and longevity of shallow cloud systems. In a recent LES study of shallow convection over the Sulu Sea in the Philippines, Yamaguchi et al. (2019) find that wind shear leads to a stronger clustering of clouds and slightly increased cloud‐base cloud fractions and diminished cloud depths. Brown (1999) shows that shear can strongly affect the surface wind via momentum transport but that it has little effect on the turbulence kinetic energy (TKE) budget, on scalar fluxes, and on cloud properties. This is in contrast to the dry convective boundary layer, where shear has a strong impact on the TKE budget (Fedorovich & Conzemius, 2008, and references therein).

The present study investigates how vertical wind shear influences trade‐wind cumulus convection, including shallow cumulus and cumulus congestus below the freezing level. For instance, we ask how shear impacts cloud tops, cloud amount, and the structure of the boundary layer. To this end, we used an idealized large‐eddy simulation (LES) case—inspired by Bellon and Stevens (2012) and Vogel et al. (2016) and not unlike the typical atmosphere in the trades—aiming at a fundamental understanding of the sensitivity to forward and backward shear (BS; by which we mean an increase and decrease, respectively, of the zonal wind speed with height) of different strengths.

The remainder of this paper is structured as follows. We first explain our idealized LES setup and the wind shear variations we impose. The results are then presented in a twofold manner. First, we discuss the effects of shear on the cloud and boundary‐layer evolution, showing results from large‐ and small‐domain simulations with interactive and prescribed surface fluxes. Second, focusing on the large‐domain runs with constant surface fluxes, we discuss how shear impacts the cloud structure and cloud depth without surface flux responses. We end with a concluding discussion and an outlook on future work. In an appendix, we discuss the influence of shear on the clouds' vertical‐velocity budget.

## 2 Experimental Design

We carried out LESs using Version 4.2 of the Dutch Atmospheric Large Eddy Simulation (DALES Heus et al., 2010). In our experimental setup, we prescribed large‐scale forcings and initial profiles typical of the North Atlantic trades at a latitude of $\varphi =15^{\circ }$N (sections 2.1–2.3). We used a domain of 50.4 × 50.4 km^2^, with a resolution of 100 m in the horizontal directions and doubly periodic boundary conditions. The domain top is at about 18 km, and the vertical grid is nonuniform: starting with 10 m at the surface and increasing by a factor of 0.01 at each level to about 190 m at the domain top. In order to evaluate the effect of different surface winds and surface heat fluxes that develop under shear, we performed simulations with interactive and prescribed sensible and latent surface fluxes (section 2.4). We also conducted simulations on a smaller domain (12.6 × 12.6 km^2^) where the development of cold pools and deeper clouds is less pronounced (Vogel et al., 2016).

### 2.1 Thermodynamics

**Figure 1:**
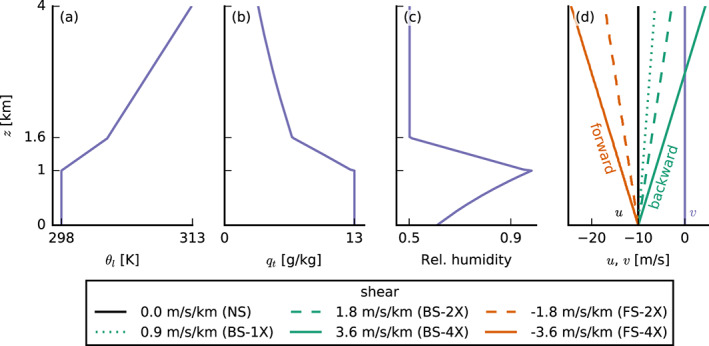
Initial profiles of (a) the liquid water potential temperature *θ*_*l*_, (b) total water specific humidity *q*_*t*_, (c) relative humidity, and (d) the two wind components *u* and *v*. Purple profiles are the same in all simulations. Orange stands for forward shear (FS) and green for backward shear (BS). Same line types indicate the same amounts of absolute shear (1X, 2X, and 4X). The color coding of the different shears is the same for all other figures.

The standard case setup is inspired by that of Vogel et al. (2016) and Bellon and Stevens (2012), who introduced an idealized modeling framework with only a limited set of parameters that represent the large‐scale flow. The initial temperature and humidity profiles of our simulations (Figure 1) have a well‐mixed layer of 1 km depth over a surface with a constant sea surface temperature (SST) of 300 K. The mixed layer is topped by a 600 m‐deep inversion layer. In the free troposphere, the profile of liquid water potential temperature *θ*_*l*_ follows a constant lapse rate of 4 K/km, and the relative humidity (RH) is constant with height at 50%. We applied a constant radiative cooling rate of −2.5 K/day to *θ*_*l*_ (i.e., no diurnal cycle), which promotes relatively strong shallow convection, allowing for the development of the congestus clouds we are interested in. Compared to Vogel et al. (2016), we increased the domain top to 18 km to allow for deeper convection. Between 10 and 18 km, the radiative cooling is quadratically reduced to zero. The RH reaches zero at about 14 km, which is also the lower boundary of the sponge layer in our LES. The *θ*_*l*_ lapse rate above 10 km is 8 K/km reflecting a stable upper atmosphere. In all simulations, we used a single‐moment ice microphysics scheme (Grabowski, 1998) and allowed for precipitation assuming a constant cloud droplet concentration of 60 cm^−3^.

### 2.2 Large‐Scale Subsidence

Different than Vogel et al. (2016), we used a weak‐temperature‐gradient (WTG) assumption to calculate the subsidence profile, as the deeper congestus clouds that develop increasingly violate the assumption of a strongly subsiding atmosphere. Practically, the WTG method was implemented following Daleu et al. (2012): Above a reference height, we calculated the subsidence rate *w*_*s*_ such that it maintains the virtual potential temperature *θ*_*v*_ close to its initial (reference) profile *θ*_*v*, 0_ according to

(1) $$w_{s}=\frac{1}{\tau }\frac{\bar{\theta_{v}}-\theta_{v,\;0}}{\partial_{z}\theta_{v,\;0}},$$

where the overbar indicates slab averaging, *∂*_*z*_ symbolizes the vertical derivative, and *τ* is the relaxation time scale, which can be thought of as the time scale over which density anomalies are redistributed by gravity waves and thus how fast the circulation acts to counteract the heating induced by convection. We set τ=1 hr, a rather short time scale that avoids the buildup of large‐density anomalies and unphysically high subsidence rates during episodes of deeper convection. WTG is not valid at levels where turbulence and convection effectively diffuse gravity waves. Therefore, we only apply WTG above 3 km, and below that (aligned with the bulk of the cloud layer above which cloud fraction becomes small), we linearly extrapolate *w*_*s*_ to zero. We also apply a nudging with a time scale of 6 hr toward the initial *q*_*t*_ (total water specific humidity) profile in the free troposphere (above 4 km) to avoid spurious moisture tendencies.

### 2.3 Winds

The winds in our simulations are subjected to a large‐scale forcing that involves only the pressure‐gradient and Coriolis forces:

(2) $${\left(\frac{du}{dt}\right)}_{ls}=fv-\frac{1}{\rho }\frac{dp}{dx}=f(v-v_{g}),$$

(3) $${\left(\frac{dv}{dt}\right)}_{ls}=-fu-\frac{1}{\rho }\frac{dp}{dy}=-f(u-u_{g}),$$

where *f* is the Coriolis parameter, *ρ* the density, *p* the pressure, and *u*_*g*_ and *v*_*g*_ are the geostrophic winds. We use initial profiles of zonal and meridional winds that are equal to the imposed geostrophic wind ( $u_{0},v_{0}=u_{g},v_{g}$). We neglect large‐scale horizontal wind advection, so that departures in the wind away from the geostrophic profiles are entirely due to the Coriolis force and the frictional force stemming from turbulence and convection. Because initially, the surface winds are in geostrophic balance, the simulation will undergo a transition toward ageostrophic surface winds (an Ekman balance). In this transition, the wind shear is effectively felt and adjusted through vertical mixing.

**Figure 2:**
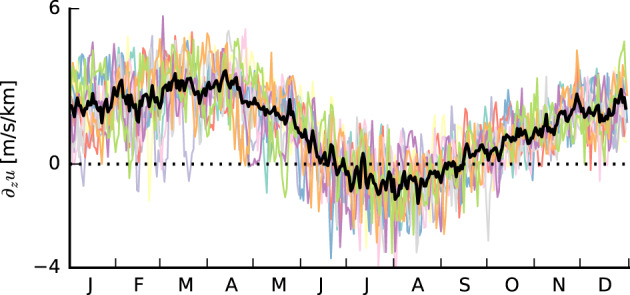
Time series of the amount of zonal shear between 1 and 3 km for the years 2008 to 2017 averaged over the area from 9° to 19°N and from 50° to 59°W (colored lines). The black line is the average over all 10 years. The dotted horizontal line indicates 0 m/(s km). Data are from the ERA5 reanalysis.

We based the wind profiles in our simulations on typical conditions in the trades, where vertical shear in the zonal wind component *u* is most common and to first order set by large‐scale meridional temperature gradients through the thermal wind relation:

(4) $$\frac{\partial u_{g}}{\partial z}≃-\frac{g}{fT}\frac{\partial T}{\partial y},$$

where *T* the temperature and *g* the gravitational constant. In the Northern Hemisphere, temperature decreases poleward (*∂*_*y*_*T* < 0), so that *∂*_*z*_*u*_*g*_ > 0, which implies that winds become increasingly westerly (eastward) with height. *∂*_*z*_*u* > 0 is indeed typical for most of the year, as derived from daily ERA5 data (12:00 UTC) from 2008 to 2017 within 9°–19°N and 50°–59°W (Figure 2). In boreal summer, when the ITCZ is located in the Northern Hemisphere and meridional temperature differences within the subtropical belts are smaller, *∂*_*z*_*u* is closer to zero or even negative. Vertical shear in the meridional wind component is close to zero year‐round (not shown).

Further analysis of daily profiles (not shown) reveals substantial day‐to‐day variability in the zonal wind profiles, regardless of the season, with reversals from negative to positive shear or zero shear from one day to the next, or vice versa. Forward shear (FS; here *∂*_*z*_*u* < 0) is to some extent a frequent feature of the atmospheric flow in the trades—not only during summer. However, BS (here *∂*_*z*_*u* > 0) is still the most common.

The magnitude of shear we imposed in our simulations is not far from what we derived from ERA5. We ran simulations with different values of zonal shear, while setting $\partial_{z}v_{g}=0$. The zonal wind profile has either no shear (NS; solid black line in Figure 1d), FS (*∂*_*z*_*u*_*g*_ < 0, orange lines), or BS (*∂*_*z*_*u*_*g*_ > 0, green lines). The FS and BS simulations have different shear strengths ranging from $|\partial_{z}u_{g}|=0.9$ ×10^−3^ s^−1^ (1X, dotted line in Figure 1d) over $|\partial_{z}u_{g}|=1.8$ ×10^−3^ s^−1^ (2X, dashed lines) to $|\partial_{z}u_{g}|=3.6$ ×10^−3^ s^−1^ (4X, solid colored lines); see also Table 1.

**Table 1:** Overview of the Various LES Experiments on a Large (50.4 × 50.4 km^2^) or Small Domain (12.6 × 12.6 km^2^) and With Interactive (Constant SST) or Fixed Surface Fluxes

			BS			FS	
	Accronym	NS	1X	2X	4X	2X	4X
Shear	[10^−3^ s^−1^]	0.0	+0.9	+1.8	+3.6	−1.8	−3.6
Large domain	interactive surface fluxes	*√*	*√*		*√*		*√*
	prescribed surface fluxes	*√*	*√*		*√*		*√*
Small domain	prescribed surface fluxes	*√*	*√*	*√*	*√*	*√*	*√*

*Note*. For each set, we differentiate between runs without wind shear (NS); runs with weak (1X), medium (2X), or strong (4X) backward (BS) shear; and runs with medium or strong forward (FS) shear (see also Figure 1d).

The response to shear is not entirely insensitive to the choice of advection scheme. Here, scalar and momentum advection was performed using a fifth‐order advection scheme in the horizontal direction and a second‐order advection scheme in the vertical direction. Using a second‐order scheme in the horizontal further increased the differences among the shear cases (in particular under free surface fluxes), which we attribute to the fact that the second‐order scheme accumulates a lot of energy on the smallest length scales close to the grid size. To reduce horizontal advective errors and allow for a larger time step, the grid was horizontally translated using a velocity that is equal to the imposed wind at 3 km height (Galilean transform; see, e.g., Wyant et al., 2018).

### 2.4 Surface Fluxes

The control simulations were run for 2 days with interactive surface fluxes, which are parametrized using standard bulk flux formulae:

(5) $${\left(\psi w\right)}_{s}=-C_{S}U_{1}(\psi_{1}-\psi_{s}),$$

(6) $$u_{*}=\sqrt{C_{M}}U_{1},$$

where $\psi \in \left\{q_{t},\theta_{l}\right\}$, *U* is the wind speed, *u*_∗_ the surface friction velocity, and the subscripts *s* and 1 stand for the surface values and values on the first model level, respectively. The constants *C*_*S*_ and *C*_*M*_ are the drag coefficients, and they depend on the stability and on the scalar and momentum roughness lengths, which we both set to $z_{0}=1.6\times 10^{-4}$ m. The drag coefficients are computed following Monin‐Obukhov similarity theory (as described in Heus et al., 2010). Additionally, a set of experiments was conducted in which the surface fluxes were kept constant.

## 3 Impact of Shear on Cloud‐ and Boundary‐Layer Evolution

We first focus on the differences in cloud and boundary‐layer structure that have developed by the end of a 2‐day simulation, using 12‐hourly averaged profiles (Hours 36–48), unless noted otherwise.

### 3.1 Interactive Surface Fluxes

**Figure 3:**
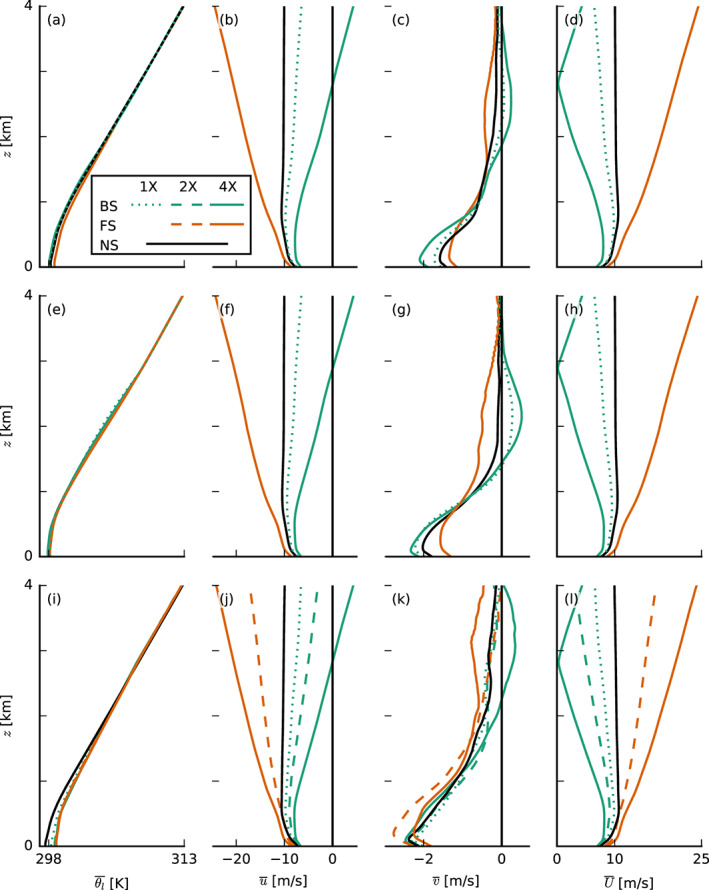
Slab‐averaged profiles of thermodynamic quantities of the large‐domain simulations with interactive surface fluxes (top row, a–d), with prescribed surface fluxes (middle row, e–h) and small‐domain simulations (bottom row, i–l). Shown are averages over the last 12 hr of each simulation of (a, e, and i) the liquid water potential temperature *θ*_*l*_ and (b, f, and j) zonal, (c, g, and k) meridional, and (d, h, and l) total wind speed, *u*, *v*, and *U*, respectively. The line colors and types are explained in Figure 1 and are the same in all following figures.

**Figure 4:**
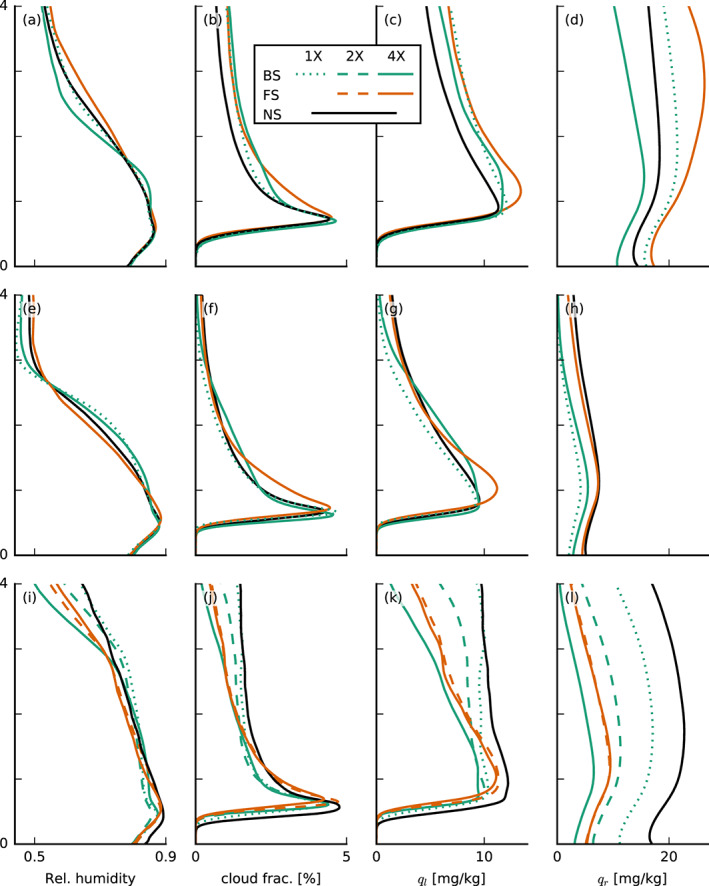
Slab‐averaged profiles of thermodynamic quantities of the large‐domain simulations with interactive surface fluxes (top row, a–d), with prescribed surface fluxes (middle row, e–h) and small‐domain simulations (bottom row, i–l). Shown are averages over the last 12 hr of each simulation of (a, e, and i) the relative humidity, (b, f, and j) cloud fraction, (c, g, and k) liquid water specific humidity *q*_*l*_, and (d, h, and l) rain water specific humidity *q*_*r*_.

Similar to the findings of Brown (1999), who ran simulations for different wind shear on a very small domain (6.4 × 6.4 km^2^), the influence of shear (Figures 3b–3d) on the thermodynamic structure of the boundary layer is overall marginal (Figures 3a and 3b), but nonetheless evident in the RH, cloud fraction, liquid water, and rain water profiles (Figures 4a–4d). In the presence of shear, regardless of its direction, cloud fractions above cloud base (approximately 700 m) are larger. In the FS‐4X case the layer above 2 km is notably moister, whereas the BS‐4X case has a more pronounced decrease of RH (which we interpret as the boundary‐layer top) around 2 km. From strong backward to strong FS, we thus observe a deepening of the moist layer and the disappearance of a pronounced hydrolapse.

**Figure 5:**
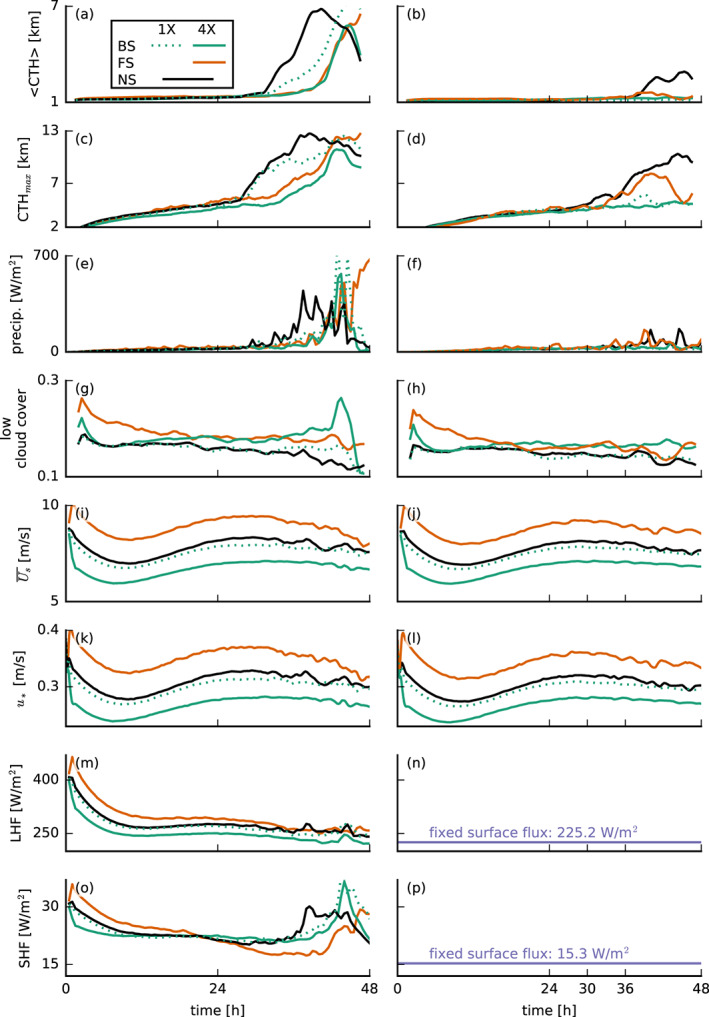
Time series of (a, b) the average and (c, d) the maximum cloud‐top height (CTH), (e, f) the surface precipitation flux, (g, h) the low cloud cover (*z* < 4 km), (i, j) the domain‐averaged total wind speed at 5 m height *U*_*s*_, (k, l) the surface friction velocity *u*_∗_, (m, n) the surface latent heat flux *LHF*, and (o, p) the surface sensible heat flux *SHF* for the interactive‐ (left column) and prescribed‐surface‐flux simulations (right column).

Differences in the depth of convection are best seen from the rain water profiles (Figure 4d) as well as the time series of average and maximum cloud‐top heights (CTHs), surface precipitation, and low cloud cover, defined as the projected cloud amount from heights up to 4 km (Figures 5a, 5c, 5e, and 5g). Differences in cloud tops start to be pronounced only on the second day of the simulations, but looking closer, one can see that the highest cloud tops on Day 1 are those of the FS‐4X simulations (in orange). On Day 2, the NS simulation develops the deepest clouds with even an average cloud top near 7 km, whereas clouds in the simulations with shear, regardless of its sign, remain shallower and rain less. During the final 12 hr, clouds in all simulations show a pronounced deepening, and the FS‐4X case even develops deeper clouds than the NS case, as well as more rain. Because we only use a simple single‐moment ice microphysics scheme here, we are cautious with the interpretation of the cloud field when it deepens beyond the freezing level. Instead, we wish to focus on the deepening from shallow cumuli to congestus with tops near 4 km. Apparently, shear plays a role at hindering that development, in particular under BS.

Figure 5 shows that the surface heat fluxes play a key role in the deepening responses. Heat fluxes diverge very early on in the simulations, whereby the largest and smallest fluxes develop for the FS‐4X and BS‐4X cases, respectively (Figures 5m and 5o). This exemplifies an important and perhaps often overlooked influence of wind shear. Given the same constant (geostrophic) forcing at the surface, a difference in zonal wind speeds can develop at the surface, due to the different zonal wind shear, which is felt near the surface through turbulent mixing, at first, and then also through the Coriolis force as the wind starts to turn (see Equation 2 and Figures 3b and 3c). These differences in surface winds (Figure 5i) give rise to the differences in surface fluxes (see Equation 5).

As clouds deepen in all simulations during Day 2, the difference in surface heat fluxes becomes smaller, as downward mixing of warm and dry free tropospheric air reduces the surface sensible heat flux while promoting the latent heat flux (Nuijens & Stevens, 2012). The increase in the sensible heat fluxes in the final 6 hr may be attributed to precipitation and evaporative cooling of rain water in the subcloud layer (e.g., cold pools; Figure 5e).

### 3.2 Prescribed Surface Fluxes

In light of these results, an important question is whether the surface fluxes are the only factor that plays a role in the development of convection, or whether shear has other more direct effects, including on the organization of clouds. Therefore, we carried out simulations with prescribed surface heat fluxes with relatively low magnitudes (namely SHF=15.3 W m^−2^ and LHF=225.2 W m^−2^; see the right column in Figure 5 and second row in Figures 3 and 4) as to minimize the development of very deep convection. Note that the surface friction (or surface momentum flux) is unchanged (Figures 5k and 5l).

Apparently, the sensitivity of cloud deepening to shear does not change its overall character when we prescribe the surface heat fluxes. Clouds are overall shallower with lower cloud fractions above 1 km (Figures  4f, 5b, and 5d), because the prescribed surface fluxes are smaller than in the interactive flux runs. But the FS‐4X case still develops the largest relative humidities above the boundary layer (>2.5 km), whereas the BS‐4X case has the most pronounced hydrolapse near the boundary‐layer top (Figure 4e). Again the FS‐4X case tends to produce somewhat deeper clouds during Day 1 but falls behind the NS case on Day 2. The BS‐4X and BS‐1X cases remain even shallower.

**Figure 6:**
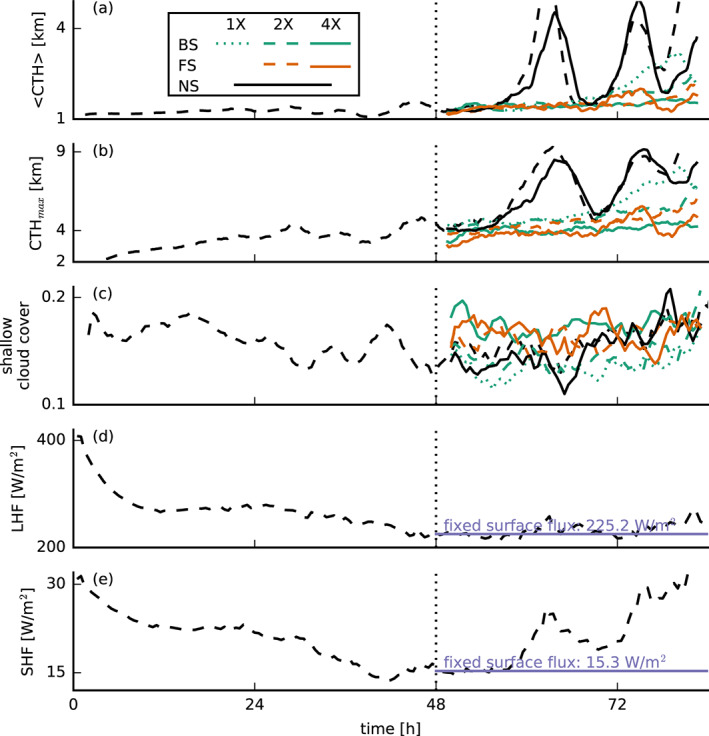
Time series of (a) the average and (b) the maximum cloud‐top heights (CTH), (c) the low cloud cover (*z* < 4 km), and the (d) surface latent and (e) surface sensible heat fluxes for the small‐domain simulations (48–84 hr). In addition to the standard line types (see Figure 1), the dashed black lines indicate a nonsheared simulation with interactive surface fluxes that is used to initialize the simulations at t=48 hr by perturbing the wind profiles and fixing the surface fluxes.

From previous studies (e.g., Malkus, 1949; Neggers et al., 2003; Yamaguchi et al., 2019) it is known that shear tilts clouds and thus increases cloud cover. In our FS and BS simulations, the tilt occurs in the negative and positive *x* directions, respectively, which enhances the low cloud cover by 10–20% (Figures 5g and 5h). A similar increase develops within a short time also after instantaneously introducing shear into a previously nonsheared system (Figure 6c; discussed below). Besides this expected impact on cloud cover, there are also some small differences in the cloud fraction profiles—including near cloud base, whose sensitivity has received much attention in recent climate studies (e.g., Bony et al., 2017; Vial et al., 2017). In the presence of shear, we observe a slightly larger maximum cloud fraction near cloud base (500–700 m) in the simulations with prescribed surface heat fluxes (Figures 4b and 4f), in line with previous studies (e.g., Brown, 1999; Yamaguchi et al., 2019). BS‐4X has a higher *q*_*t*_ variance at these heights, which are due to a few percent more active cloud (not shown) and which could explain the higher cloud fraction. In the FS‐4X case, the larger cloud‐base cloud fraction is explained by more passive cloud (not shown).

### 3.3 Sensitivity Tests on a Smaller Domain

The same difference in deepening between the shear cases can be observed when applying instantaneous perturbations to the (geostrophic) wind shear, while keeping the surface fluxes constant (Figure 6). In these sensitivity tests, carried out on a 16‐fold smaller domain (see Table 1, which is still four times as large as the one used by Brown, 1999), we start from the equilibrium state of the NS case after 2 days and then apply a perturbation. We then let the system evolve for another 36 hr. Also, here it is evident that when wind shear is introduced, convective deepening is prevented (Figures 6a and 6b) in comparison with how the simulation develops without a perturbation (dashed black line in Figure 6). Even very weak shear (BS‐1X, dashed green line) can effectively reduce the clouds' depth and delay cloud deepening.

It is worthwhile to compare the profiles of RH and cloud fraction on the small domain (Figures 3i–3l and 4i–4l) with those on the large domain. The 16‐fold smaller domain leads to much higher relative humidities and cloud fractions above 2 km. This can be explained by the lack of spatial organization of shallow convection on the small domain. Increasing the domain size generally tends to organize the shallow convection into deeper and larger clusters, which leads to a shallower, warmer, and drier domain. Vogel et al. (2016) found that on a larger domain the likelihood of developing a strong updraft and deep cloud increases and that larger domains support stronger and deeper updrafts by allowing them to spread their compensating subsidence over a larger area. In the absence of spatial organization on the small domain, we can observe that only the FS‐4X case behaves differently compared to the large domain. This case is no longer comparably moist or even moister than the NS case and its cloud fraction, and RH profile is now more in line with that of the BS‐4X case. This hints at a role of spatial organization in explaining the response to FS, which we address later.

**Figure 7:**
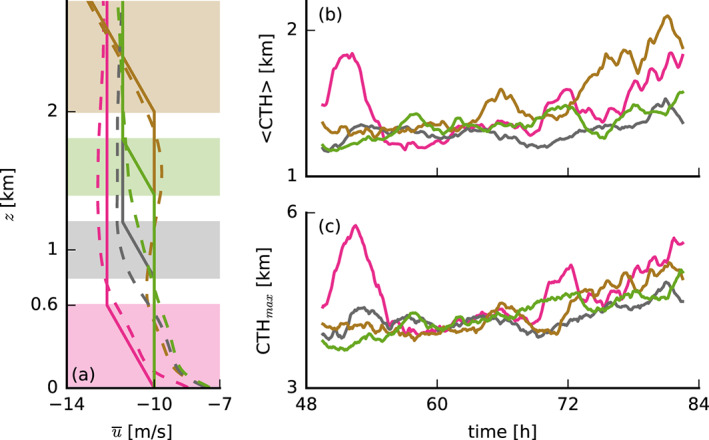
(a) Initial (solid lines) and slab‐averaged profiles (from the last 12 hr; dashed lines) of the zonal wind *u* of simulations in which shear is only applied at limited height levels, as well as (b, c) the corresponding time series of the (b) average and (c) maximum cloud‐top heights. Pink lines depict FS‐4X shear at 0–0.6 km, gray at 0.8–1.2 km, green at 1.4–1.8 km, and brown at 2–10 km.

Using the same experimental setup (i.e., small domain, fixed surface fluxes, and sudden perturbation of the wind profile), we carried out some further sensitivity tests in which we applied FS to specific layers (Figure 7). These simulations show that shear is particularly effective at keeping convection shallow when applied in the lower cloud layer (gray and green lines in Figure 7), whereas shear in the subcloud layer (pink) or near cloud tops (brown) still leads to cloud deepening.

## 4 Sensitivity of Convective Deepening to Shear

Overall, the previous section has shown that the presence of even weak BS effectively inhibits convective deepening, while FS only slightly weakens the potential to develop deeper clouds: This inhibition reveals itself as a delay (if surface feedbacks are present) or as a complete suppression of deepening (if surface heat fluxes are fixed). On a smaller domain, FS has the same strong inhibitive effect as BS. If not through a surface flux response, what is the mechanism through which BS oppresses convection, while FS seems to allow for cloud deepening (on a sufficiently large domain)? Two hypotheses, borrowed from studies of deep convection, are as follows:

1. Wind shear changes the rate of entrainment, the updraft buoyancy, and updraft speed: As clouds get tilted through any absolute amount of shear, they may suffer from more lateral entrainment and opposing pressure perturbations that limit updraft speeds and cloud vertical extent.
2. Wind shear changes the structure and organization of shallow cloud systems. For instance, FS helps to separate regions of updrafts and downdrafts and may therefore sustain larger subcloud circulations that continue to feed moisture into already cloudy areas. FS may also interact with cold‐pool fronts to force stronger updrafts.

To investigate these ideas, we consider only the simulations with prescribed surface fluxes and focus on the period between 30 and 36 hr (unless noted otherwise). In this period, clouds first start to deepen from shallow cumulus to congestus at different rates depending on shear, and the cloud field has not developed deep convection yet (cf. Figures 5b and 5d).

### 4.1 Entrainment and Updraft Speeds

**Figure 8:**
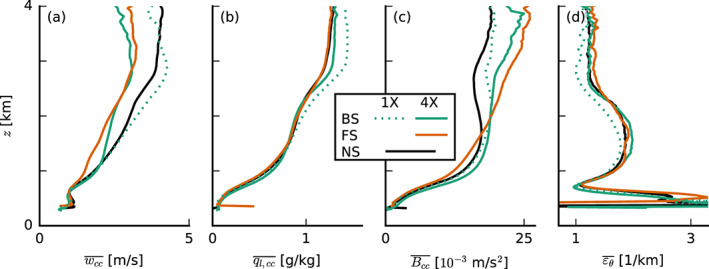
Slab‐averaged profiles of (a) the cloud‐core vertical velocity *w*_*cc*_, (b) the cloud‐core liquid water specific humidity *q*_*l*, *cc*_, (c) the cloud‐core buoyancy *B*_*cc*_, and (d) the fractional entrainment rate *ε*_*θ*_ of *θ*_*l*_ (averaged from 30 to 36 hr of the simulations with prescribed surface fluxes).

The FS‐4X and BS‐4X cases have significantly lower updraft speeds in the cloud cores (*q*_*l*_ > 0 and $\theta_{v}^{\prime }>0$) compared to the NS and BS‐1X cases (Figure 8a), which appears key to explaining the lower cloud‐top heights that develop under shear. However, the strongly sheared simulations contain nearly the same amount of cloud‐core liquid water and are notably more buoyant, especially above 2 km (Figures 8b and 8c). A similar picture is established if we sample on cloudy points (*q*_*l*_ > 0). Furthermore, the vertical mass flux is hardly affected by shear (not shown), as also found by Neggers et al. (2003). Buoyancy itself is evidently not key to explaining the weaker updrafts under shear (although it likely explains the stronger updrafts below 1 km in the BS‐4X case). The relatively low buoyancy in cloud cores of the NS case (at least above 2 km) is because the environment surrounding the nonsheared clouds is warmer in terms of *θ*_*v*_ (not shown), because clouds in that simulation are already mixing across a deeper layer (Figure 5d), while the clouds themselves have a similar *θ*_*v*_ in each case. Vogel et al. (2016) also showed how quickly the thermodynamic structure of the boundary layer changes as shallow cumuli develop into cumulus congestus.

Using the simple entraining plume model by Betts (1975) to calculate the fractional entrainment rate *ε*_*θ*_ of *θ*_*l*_ (Figure 8d), we find that clouds in the BS and FS cases entrain only marginally more environmental air than in the NS case if anything (also if we consider entrainment of *q*_*t*_; not shown). This suggests that there is no larger lateral entrainment due to shear that could explain weaker vertical development. We also find that lateral entrainment plays a relatively small role in the conditionally sampled vertical‐velocity budget (Appendix A).

The weaker cloud‐core vertical velocities under shear are in line with studies of deep convection in squall lines, in particular the recent study by Peters et al. (2019) and earlier work by similar authors (Parker, 2010; Peters, 2016), who show that slanted updrafts are weaker than upright ones. Peters et al. (2019) decompose the vertical momentum equation into four terms that describe the processes that regulate the vertical acceleration of updrafts: (1) a term associated with momentum entrainment and detrainment, (2) a (downward oriented) dynamic pressure acceleration term, (3) a (downward oriented) buoyancy pressure acceleration term, and (4) a buoyancy acceleration term (which includes the entrainment of thermodynamic properties that can limit updraft buoyancy). They show that shear mostly enhances the dynamic pressure perturbations, which can be interpreted as an aerodynamic lift force due to the shear‐driven crossflow (perpendicular to the direction of ascent). Unlike the lift associated with aircraft wings, the lift in slanted thermals experiencing crossflow is directed downward. A handful of studies on the vertical‐velocity budget of shallow convection have also noted a minor role of entrainment in explaining updraft speeds (e.g., de Roode et al., 2012; Morrison & Peters, 2018; Romps & Charn, 2015; Tian et al., 2019).

An investigation of the vertical‐velocity budget—a subject on its own as demonstrated by the aforementioned studies—goes beyond our goal, but we can get an impression of the importance of the pressure perturbations by sampling the vertical‐velocity budget in cloudy updrafts, following de Roode et al. (2012), here included in Appendix A. We find that differences that contribute to the vertical velocity in the cloud layer are predominantly found in the pressure‐gradient and buoyancy terms, whereas differences in the horizontal flux of resolved and subgrid vertical momentum across the cloud boundaries (e.g., entrainment) are only important near cloud base (<1 km) where other tendencies are small. Near cloud tops (>2 km), updrafts in the sheared runs experience a larger negative pressure‐gradient force. A quick look at the total pressure perturbations in *x*‐*z* cross sections also confirms that pressure perturbations, especially near the slanted sides and tops of the clouds, are more pronounced under shear (not shown).

Overall, our results emphasize that shear keeps clouds shallower by weakening updrafts. However, we also observe that clouds under FS have a tendency to get deeper than under BS. This is explored next.

### 4.2 Structure and Organization of Turbulence and Clouds

**Figure 9:**
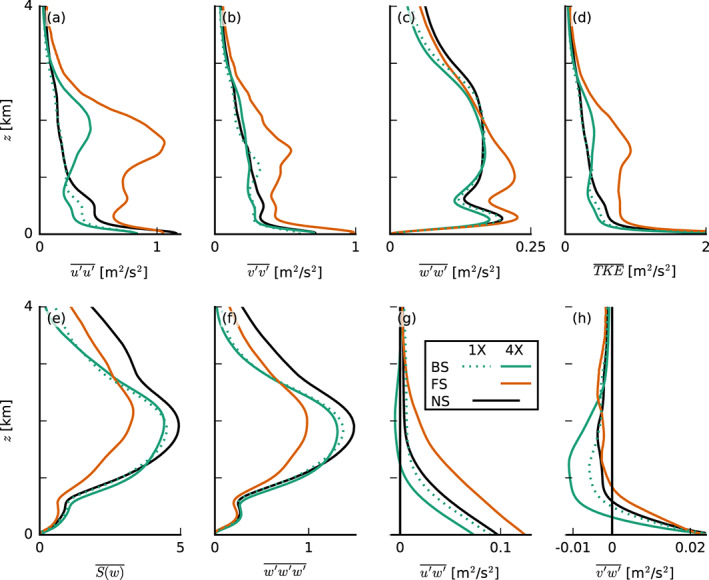
Slab‐averaged profiles of the resolved variances of (a) the zonal wind speed *u′u′*, (b) the meridional wind speed *v*′*v*′ and (c) the vertical velocity *w′w′*, (d) the turbulence kinetic energy (TKE), (e) the skewness *S*(*w*), (f) the third moment *w′w′w′* of the vertical velocity, and (g) the zonal and (h) the meridional momentum fluxes, *u′w′* and *v′w′*, respectively (averaged from 30 to 36 hr of the simulations with prescribed surface fluxes).

In Figure 9 we show a number of quantities that reveal changes to the character of the turbulence structure of the boundary layer: the domain‐averaged variances of the velocity components, the TKE, the skewness *S* and third central moment of the vertical velocity $\bar{{w{}^{\prime }}^{3}}$, and finally the zonal and meridional momentum fluxes. Velocity variances are clearly enhanced in the FS‐4X case, where the vertical gradient in wind speed between the surface and cloud tops—the shear—is largest (cf. Figures 3f–3h). Consequently, TKE and the momentum fluxes are larger, in agreement with Brown (1999). Momentum fluxes at the surface are also largest for the FS‐4X case, leading to a larger surface friction (see also Figure 5i and 5j) and larger surface‐layer shear.

Several authors have noted that convection can transition from a closed‐cell structure to roll structures due to shear (e.g., Khanna & Brasseur, 1998; Salesky et al., 2017; Sykes & Henn, 1989). A parameter that controls this transition is the ratio of the surface friction velocity *u*_∗_ to the convective velocity scale *w*_∗_ (Sykes & Henn, 1989) or equivalently the ratio of the Obukhov length and the boundary‐layer height. While the exact value of *u*_∗_/*w*_∗_ at which the transition takes place depends on other properties of the flow (different studies report values between 0.27 and 0.65), low values are clearly associated with cellular convection and high values with roll structures (Fedorovich & Conzemius, 2008; Salesky et al., 2017). In our simulations, *u*_∗_/*w*_∗_ has rather low values, which do not differ greatly among the various shear cases (ranging from about 0.30 for BS‐4X to 0.37 for FS‐4X), indicating that convection is mainly buoyancy driven and not shear driven in all our simulations.

**Figure 10:**
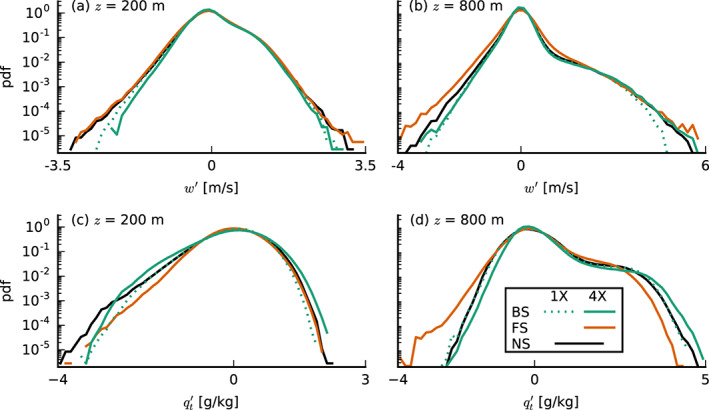
Probability density functions of the vertical velocity *w* (top) and the total water specific humidity deviations $q_{t}^{\prime }$ (bottom) at constant heights of (left) z=200 m and (right) z=800 m (averaged from 30 to 36 hr of the simulations with prescribed surface fluxes).

The skewness of the vertical velocity $S(w)=\bar{{w{}^{\prime }}^{3}}/{\bar{{w{}^{\prime }}^{2}}}^{\frac{2}{3}}$, which is a measure for the asymmetry of the vertical velocity distribution, is reduced with FS. This is primarily caused by the reduction in the advection of vertical velocity variance, $\bar{{w{}^{\prime }}^{3}}$, due to on average weaker updrafts into the cloud layer (Figure 8a). The variance of *w* instead is larger under FS‐4X (Figure 9c). Although the PDFs of *w* at 200 and 800 m (near cloud base) in Figures 10a and 10b are overall very similar, the FS‐4X case has notably stronger updrafts as well as stronger downdrafts (tails of the PDF). This might be a signature of the downdrafts being separated from the updraft regions. Because the FS‐4X case also has the largest absolute amount of wind shear across the subcloud layer, it has the largest positive (anticlockwise) vorticity. These results suggest that instead of narrow updrafts closely surrounded by subsidence, the FS‐4X case develops stronger ascent and descent in separated branches of a circulation that enhances moisture transport into cloudy areas.

**Figure 11:**
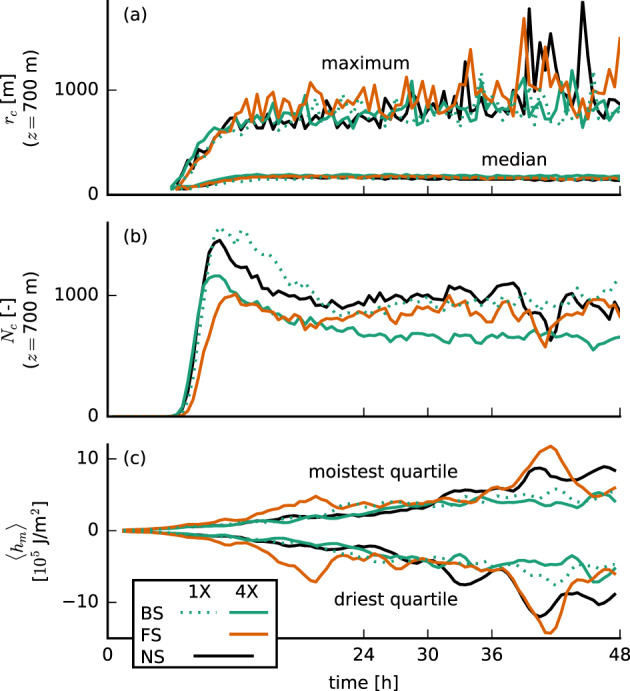
Time series of (a) the median and maximum cloud radius *r*_*c*_ at z=700 m, (b) the number of clouds *N*_*c*_ at that height, and (c) the vertically integrated moist static energy anomalies ⟨*h*_*m*_⟩ in the moistest and the driest quartiles of 12.6 × 12.6 km^2^ blocks for the simulations with prescribed surface fluxes.

Indeed, the FS‐4X case has the largest amount of domain‐averaged liquid water and cloud fraction between 800 m and 1.5 km on both small and large domains (Figures 4f, 4g, 4j, and 4k) and larger relative humidities just above cloud base (Figures 4e and 4i), even though cloud base is on average higher than for the BS and NS cases. By analyzing the mean and maximum cloud radii and the number of clouds, we also find that the FS‐4X case develops the fewest but the largest clouds (Figures 11a and 11b), whereas the NS case has more numerous smaller clouds, similar to findings by Yamaguchi et al. (2019).

**Figure 12:**
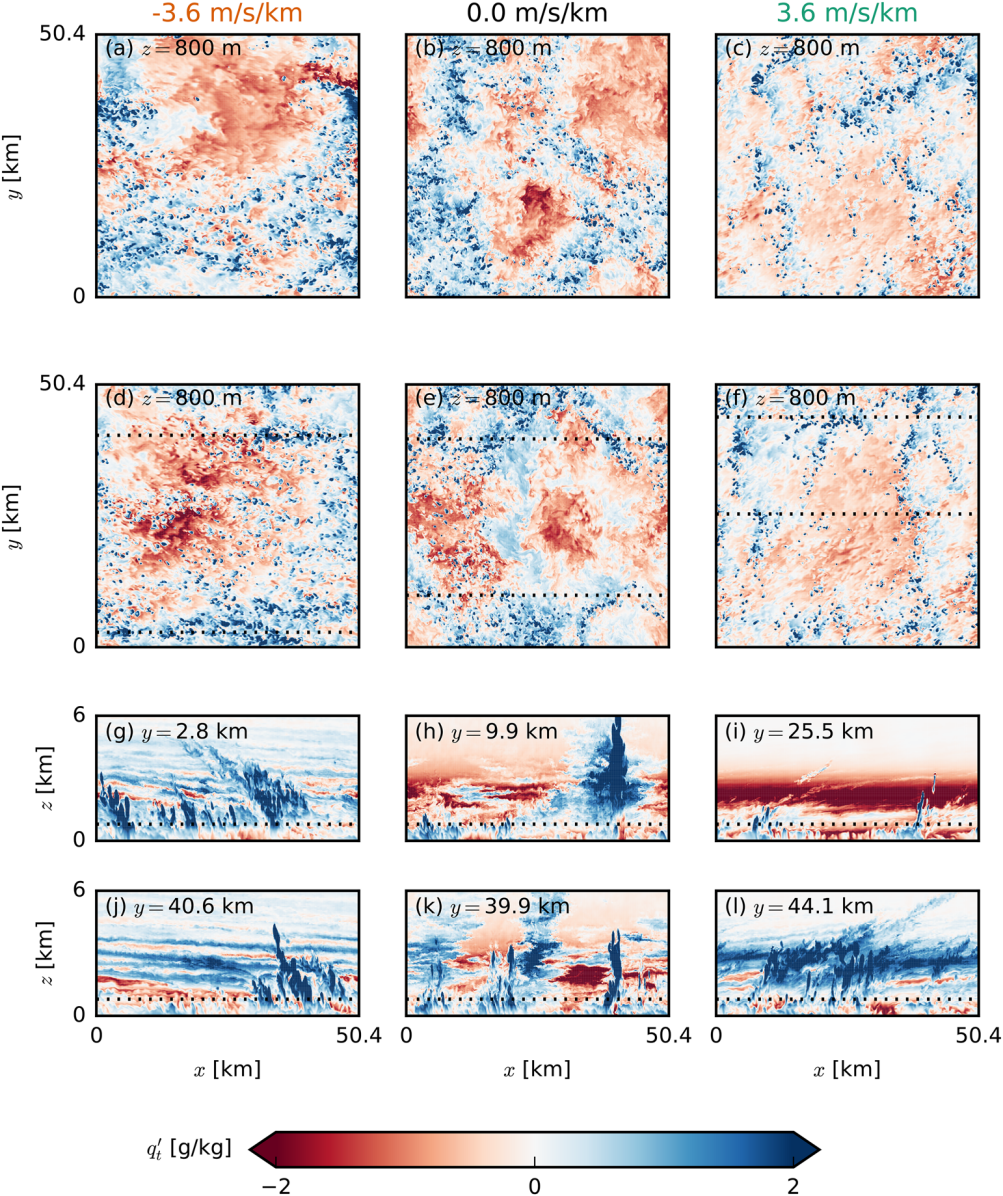
Snapshots of the LES domains of FS‐4X (left), NS (center) and BS‐4X (right) exhibiting typical characteristics in the late stages of the simulations with prescribed surface fluxes. The top two rows (a–f) show horizontal *x*‐*y* cross sections at two times ( t=39.0 hr and t=46.5 hr) near cloud base ( z=800 m) of the deviations from the mean of the total water specific humidity $q_{t}^{\prime }$. The bottom two rows (g–l) show corresponding vertical *x*‐*z* cross sections from the lowest 6 km of the domain of the latter of the two times (d–f). The horizontal dotted lines indicate the position of the respective other cross sections.

The formation or aggregation of larger clouds is also evident from the moisture field. Figure 11c shows deviations of the vertically integrated moist static energy within blocks of 12.6 × 12.6 km^2^ compared to the domain mean and compares the moistest and the driest quartiles of the domain (in terms of total water path), which is a common measure for self‐aggregation (Bretherton & Blossey, 2017). This reveals that during the first 24 hr, the strongest moistening of the moist regions and strongest drying of the dry regions take place in the FS‐4X cases. Furthermore, snapshots of the moisture field (Figure 12) show that large patches of high or low moisture are less common in the simulations with BS compared to the other cases.

**Figure 13:**
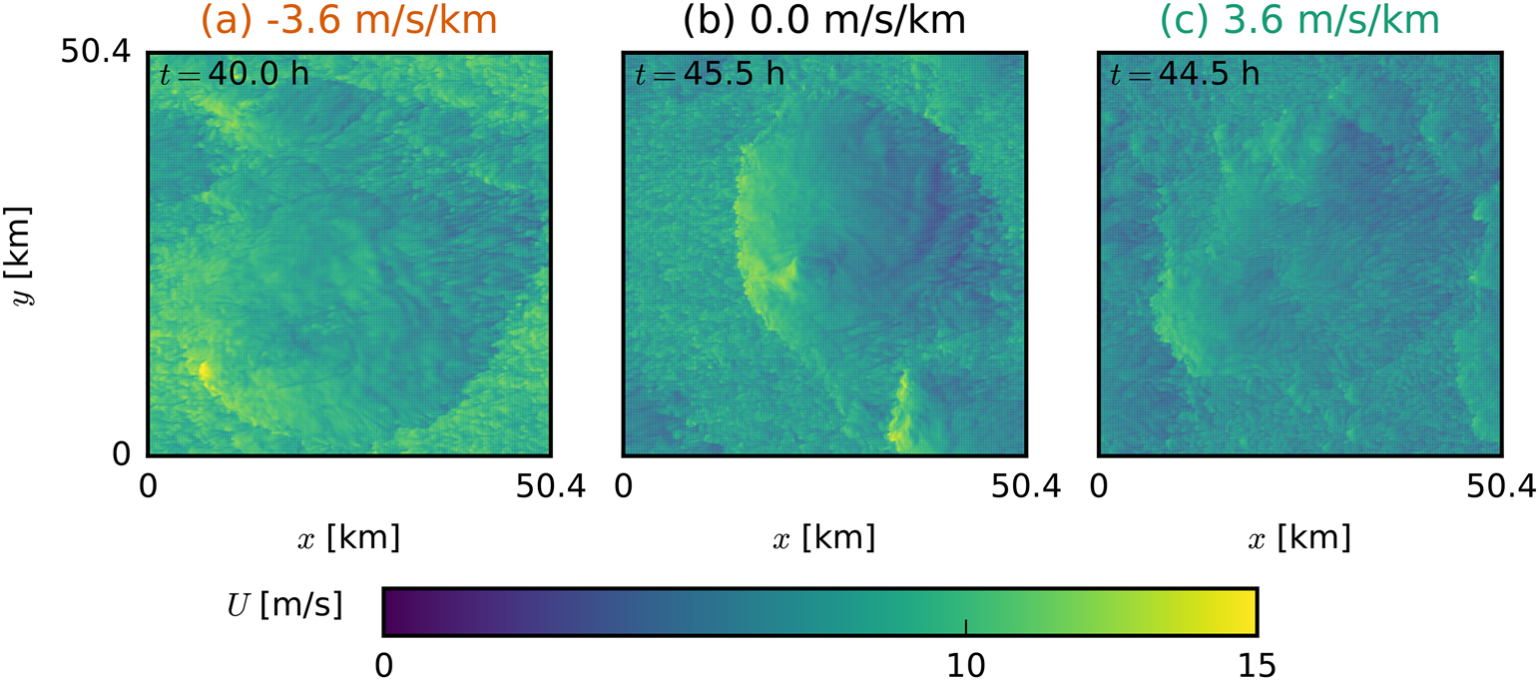
Snapshots of the LES domains of (a) FS‐4X, (b) NS, and (c) BS‐4X exhibiting typical characteristics of the total wind speed *U* in the late stages of the simulations with prescribed surface fluxes. Shown are horizontal *x*‐*y* cross sections at z=5 m.

After the first day of simulation when precipitation increases, cold‐pool effects might play an additional role in organizing the cloud and moisture field. The cold‐pool boundaries may interact with the environmental shear in the subcloud layer to trigger stronger force‐lifted updrafts under FS (e.g., Li et al., 2014). The FS and BS cases also have a different wind speed distribution within the cold pools (Figure 13). Whereas the BS case reveals the typical diverging flow with a strong easterly current left from the cold pool center and relatively stronger westerly winds toward the right, the FS case has much stronger easterly winds throughout. This may signify a role of downward momentum transport as well. The role of cold pool‐shear interaction is the subject of a follow‐up study.

## 5 Conclusions

In this paper, we have used idealized LESs initialized and forced with a geostrophic wind that is equal at the surface but has a different vertical profile (vertical wind shear). We showed that vertical wind shear influences the depth and characteristics of shallow cumulus convection and thereby the depth and structure of the trade‐wind layer. Even weak vertical shear in the zonal wind component can retard the growth of cumulus clouds, in particular when the shear vector is directed against the mean wind direction (BS). Furthermore, we have shown that shear increases the cloud fraction—an effect that has been of major interest in recent climate studies (e.g., Bony et al., 2017; Vial et al., 2017).

BS, whereby surface easterlies become upper westerlies, is typical for the winter trades, presumably because this season has a larger meridional temperature gradient between the equator and subtropics. Simulations with interactive surface fluxes reveal that BS can slow down vertical cloud development. Under BS, mean cloud tops remain near 2 km for at least 36 hr of simulation, at which point the simulations without (imposed) shear have developed clouds with mean tops near 7 km. Given the same geostrophic wind forcing at the surface, and in absence of horizontal wind advection, the weakest surface winds develop under BS. When initializing the simulations with surface winds in geostrophic balance, and no horizontal wind advection is applied, the weakest surface winds are reached under BS as the simulation approaches an Ekman balance: Relatively weaker wind speeds are then mixed toward the surface, compared to the simulations with FS or NS.

Weak shear and FS (easterlies become stronger with height) are not uncommon during boreal winter, even if they are more typical for boreal summer when the ITCZ and deep convection shift northward. The vertical development of clouds under FS is also delayed, but not as much as with BS, because simulations with FS develop the strongest surface winds and (initially) the largest surface heat fluxes.

To elucidate more direct effects of vertical shear, we repeated the simulations with prescribed surface heat fluxes. These show that the presence of shear in the cloud layer, regardless of its sign, limits updraft speeds, in line with studies of deep convection that have shown shear to inhibit convective development (e.g., Peters et al., 2019). Entrainment appears to play a minor role in setting the weaker updrafts (e.g., de Roode et al., 2012; Morrison & Peters, 2018; Romps & Charn, 2015; Tian et al., 2019). Instead, larger downward oriented pressure perturbations under both forward and BS appear to weaken vertical accelerations.

In addition, shear changes the turbulence structure of the subcloud layer. Though our simulations remain buoyancy‐driven and do not develop roll structures or cloud streets, FS develops stronger updrafts and downdrafts, a moister layer near cloud base with larger cloud fraction, fewer but larger cloud clusters, and more moisture aggregation. FS maintains the largest absolute amount of shear in the subcloud layer, which leads to a larger background vorticity and separates regions with updrafts from regions with downdrafts. This may develop a stronger subcloud circulation with sustained regions of ascending motion that feed moisture into areas of clouds. The larger cloud clusters can become deeper, as they do in the first day of simulation under FS, but are ultimately limited by weaker updraft speeds.

As clouds remain shallower under BS, the moistening of the cloud layer is more pronounced, and the top of the cloud layer is marked by a steeper decrease in humidity, as is typical near the trade‐wind inversion (e.g., Riehl et al., 1951). The moister subcloud and cloud layer, as well as a stronger inversion, will lead to more cloudiness. Therefore, we may argue that the trade winds themselves help to set the trade‐wind inversion and thus that BS is a crucial ingredient in defining the typical trade‐wind‐layer structure.

## Figures and Tables

**Figure A1:**
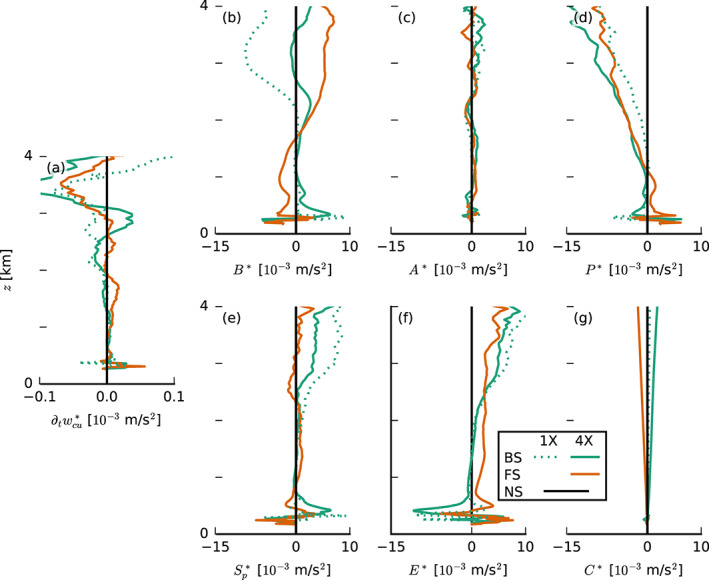
Slab‐averaged profiles (averaged from 30 to 36 hr of the simulations with prescribed surfaces fluxes) of the terms of the cloudy‐updraft vertical‐velocity budget (Equation A1) plotted as differences from the NS case (indicated by the asterisks).

## Data Availability

The exact version of the code and the input files used in this work are available online (via https://doi.org/10.5281/zenodo.4138940).

## Appendix A Impact of Shear on the Vertical‐Velocity Budget

### A.1

To study a difference in the forcing acting on the vertical velocity of cloudy updrafts in simulations with and without shear, we follow the method by de Roode et al. (2012), who applied the top‐hat approach by Siebesma and Cuijpers (1995) to compute the conditionally sampled vertical‐velocity budget in DALES:

(A1) $$\frac{\partial w_{c}}{\partial t}=\underset{B}{\underbrace{\frac{g(\theta_{v,c}-\bar{\theta_{v}})}{\theta_{0}}}}\underset{P}{\underbrace{-{\left[\frac{\partial \pi }{\partial z}\right]}_{c}}}\underset{C}{\underbrace{+2𝛀\cos\varphi u_{c}}}\underset{A}{\underbrace{-\frac{1}{2\rho }\frac{\partial w_{c}^{2}}{\partial z}}}\underset{S_{p}}{\underbrace{-\frac{1}{\rho \sigma_{c}}\frac{\partial \sigma_{c}{\bar{w^{\prime \prime }w^{\prime \prime }}}^{c}}{\partial z}}}\underset{E}{\underbrace{-\frac{\epsilon_{w}w_{c}^{2}}{1-\sigma }}},$$

where the subscript *c* stands for conditional sampling (here, on cloudy updrafts, i.e., *q*_*l*_ > 0 and *w* > 0), *g* the gravitational acceleration, *θ*_*v*_ the virtual potential temperature, *θ*_0_ a reference temperature, *π* the modified pressure, *𝛀* Earth's angular velocity, *φ* the latitude, *σ* the area fraction, *ϵ*_*w*_ the fractional entrainment rate of *w*, and *ρ* the slab‐mean density. The modified pressure *π* is defined as

(A2) $$\pi =\frac{1}{\rho }\left(p-\bar{p_{h}}\right)+\frac{2}{3}e,$$

where *p* is the pressure, *p*_*h*_ the hydrostatic pressure, and *e* the subgrid‐scale TKE. The latter is included because in DALES, $\frac{2}{3}e$ is subtracted from the subgrid momentum flux to simplify its computation; to compensate for this, the term is added back to the pressure (Heus et al., 2010). Preliminary tests show, however, that the subgrid TKE contribution to the conditionally sampled pressure term is small and insensitive to shear (not shown). The tendency on the l.h.s. of Equation A1 is calculated directly from the LES. Averaged over 6 hr (30 to 36 hr), it is close to zero. This tendency closely matches the sum of the terms on the r.h.s., which represent the buoyancy acceleration (*B*), the vertical pressure gradient (*P*), the Coriolis force (*C*), the vertical advection (*A*), the subplume vertical advection (*S*_*p*_), and the lateral entrainment *E*.

Above 1 km, in the cloud layer, the production of vertical velocity from positive buoyancy *B* is largely balanced by a sink of vertical velocity due to the pressure gradient *P*, followed by a smaller sink from advection *A*. The subplume term *S*_*p*_ is close to zero in the cloud layer, and *C* is also small (negative). The lateral entrainment term *E* is small yet positive, counter to the conventional idea that entrainment is contributing negatively to cloud updraft quantities. This unexpected sign of the diagnosed lateral entrainment rate was also observed by de Roode et al. (2012), who argued that changes in the number of sampled points as parcels enter of leave cloudy updrafts (so‐called Leibniz terms) may violate the implicit assumption that lateral entrainment is dominated by horizontal advection. As Young (1988) explained, any sampled derivative, such as of vertical velocity,

(A3) $${\left[\frac{\partial w}{\partial t}\right]}_{c}=\frac{\partial w_{c}}{\partial t}+\frac{w_{c}}{\sigma }\frac{\partial \sigma }{\partial t}+{\left\{\frac{\partial w}{\partial t}\right\}}_{L},$$

introduces an additional term that stems from Leibniz's rule of differentiation. It represents temporal changes in the sampled vertical velocity due to changes in the sampling set. To let the lateral entrainment term in Equation A1 be consistent with parametrized vertical‐velocity equations (see Equation 3 in de Roode et al., 2012), we diagnosed it as

(A4) $$-\frac{\epsilon_{w}w_{c}^{2}}{1-\sigma }=-\frac{w_{c}}{\sigma }\frac{\partial \sigma }{\partial t}-\frac{w_{c}}{\sigma }\frac{\partial M_{c}}{\partial z}-{\left[\frac{\partial u_{h}w}{\partial x_{h}}\right]}_{c}-{\left[\frac{\partial \tau_{3h}}{\partial x_{h}}\right]}_{c}-{\left\{\frac{\partial w}{\partial t}\right\}}_{L}-{\left\{\frac{\partial ww}{\partial z}\right\}}_{L}-{\left\{\frac{\partial \tau_{33}}{\partial z}\right\}}_{L},$$

where *M*_*c*_ is the mass flux. The Leibniz terms are of significant magnitude. Besides, a more complicated behavior of vertical velocity than assumed in the top‐hat approach is present (e.g., Heus & Jonker, 2008), therefore lending itself less well for estimating the fractional entrainment rate (as compared to thermodynamic quantities).

To explain how different forcings under shear can contribute to differences in the updraft speeds, Figure A1 shows these budget terms as deviations from the NS case. Positive values indicate a stronger positive contribution to updraft speed (or a smaller negative contribution). In particular, above 1 km, the FS and BS cases have a larger negative *P* contribution (Figure A1d), which is present at the same altitude where we see slower updraft speeds in the presence of shear (Figure 8a). The differences in *P* are balanced mostly by differences in *E* (in the BS‐4X case) or *B* (in the FS‐4X case). The latter results from the different development of environmental temperature and humidity, as discussed in section 4.1 and shown in Figure 8c. The NS case with its strongest updrafts develops the deepest clouds and thus the warmest boundary layer, which reduces *B*, leading to a balance in the budget over 6 hr. It thus appears that initial differences in updraft speeds develop due to differences in pressure gradients under shear, which are maintained throughout the simulation, as a balance with the buoyancy force is established.
